# Methodological Approaches for Monitoring Five Major Food Safety Hazards Affecting Food Production in the Galicia–Northern Portugal Euroregion

**DOI:** 10.3390/foods11010084

**Published:** 2021-12-29

**Authors:** Juan Rodríguez-Herrera, Ana G. Cabado, Gustavo Bodelón, Sara C. Cunha, Vânia Pinto, José O. Fernandes, Jorge Lago, Silvia Muñoz, Isabel Pastoriza-Santos, Paulo Sousa, Luís Gonçalves, Marta López-Cabo, Jorge Pérez-Juste, João Santos, Graça Minas

**Affiliations:** 1 Instituto de Investigaciones Marinas, Consejo Superior de Investigaciones Científicas (IIM-CSIC), 36208 Vigo, Spain; silviam@iim.csic.es (S.M.); marta@iim.csic.es (M.L.-C.); 2 ANFACO-CECOPESCA, Ctra. Colexio Universitario, 16, 36310 Vigo, Spain; agcabado@anfaco.es (A.G.C.); jlago@anfaco.es (J.L.); 3 CINBIO, Campus Universitario As Lagoas, Universidade de Vigo, 36310 Vigo, Spain; gbodelon@uvigo.es (G.B.); pastoriza@uvigo.es (I.P.-S.); juste@uvigo.es (J.P.-J.); 4 Galicia Sur Health Research Institute (IIS Galicia Sur), SERGAS-UVIGO, 36310 Vigo, Spain; 5 LAQV-REQUIMTE, Laboratory of Bromatology and Hidrology, Department of Chemical Sciences, Facultaty of Pharmacy, University of Porto, Jorge de Viterbo Ferreira 228, 4050-313 Porto, Portugal; sara.cunha@ff.up.pt (S.C.C.); Josefer@ff.up.pt (J.O.F.); joaolms@ff.up.pt (J.S.); 6 Center for MicroElectromechanical Systems (CMEMS-UMinho), University of Minho, 4800-058 Guimarães, Portugal; vpinto@dei.uminho.pt (V.P.); psousa@dei.uminho.pt (P.S.); lgoncalves@dei.uminho.pt (L.G.); gminas@dei.uminho.pt (G.M.)

**Keywords:** harmful algal blooms, *Listeria-monocytogenes*-containing biofilms, mycotoxins, allergens, polycyclic aromatic hydrocarbons, Galicia (Spain), Northern Portugal

## Abstract

The agri-food industry has historically determined the socioeconomic characteristics of Galicia and Northern Portugal, and it was recently identified as an area for collaboration in the Euroregion. In particular, there is a need for action to help to ensure the provision of safe and healthy foods by taking advantage of key enabling technologies. The goals of the FOODSENS project are aligned with this major objective, specifically with the development of biosensors able to monitor hazards relevant to the safety of food produced in the Euroregion. The present review addresses the state of the art of analytical methodologies and techniques—whether commercially available or in various stages of development—for monitoring food hazards, such as harmful algal blooms, mycotoxins, *Listeria monocytogenes*, allergens, and polycyclic aromatic hydrocarbons. We discuss the pros and cons of these methodologies and techniques and address lines of research for point-of-care detection. Accordingly, the development of miniaturized automated monitoring strategies is considered a priority in terms of health and economic interest, with a significant impact in several areas, such as food safety, water quality, pollution control, and public health. Finally, we present potential market opportunities that could result from the availability of rapid and reliable commercial methodologies.

## 1. Introduction

**Figure 1:**
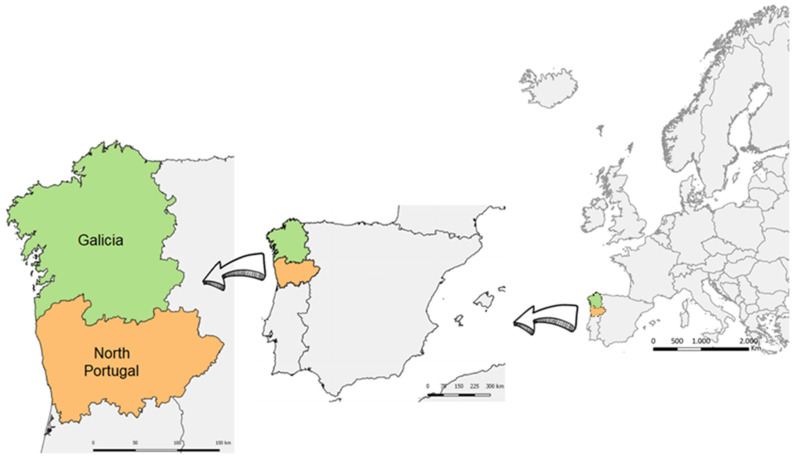
Location of the Galicia–Northern Portugal Euroregion.

Agriculture, livestock farming and fisheries (including aquaculture), and the food industry are highly important socioeconomic pillars in the Galicia–North Portugal Euroregion (Figure 1). More than 100,000 people were employed in the agri-food sector in Galicia in 2018, representing approximately 10% of total employment, with a gross added value of over EUR 4000 million [1]. In Northern Portugal, the turnover of agri-environmental and food systems exceeded EUR 4000 million and was associated with more than 60,000 jobs [2]. A wide variety of foods, particularly wine, maize, seafood, and dairy and meat products, are commonly produced in the Euroregion. In particular, the production of seafood in Galicia is the highest of all the regions in the European Union (EU) [3]. Accordingly, the Smart Specialization Strategy document identified the food industry as an area for collaboration in the Galicia–North Portugal Euroregion [4]. In particular, there is a need for action to help to ensure the provision of safe and healthy foods through the application of biotechnology and other key enabling technologies. Following this objective, the FOODSENS project [5] is aimed at developing biosensing devices to detect several hazardous chemical and microbiological agents of high relevance for the safety of food produced in the Euroregion, specifically those associated with harmful algal blooms (HABs), mycotoxins, *Listeriamonocytogenes*-containing biofilms, allergens, and polycyclic aromatic hydrocarbons (PHAs). Maximum permitted levels and official control measurements for each of these hazards are established in different EU Commission regulations applicable in both Spain and Portugal.

Galicia is the largest bivalve-producing area in the EU, and Portugal has a strategic plan that aims to achieve a 300% increase in aquaculture production, up to ca. 35,000 t by 2023 [6]. However, production areas are occasionally affected by HABs, which produce toxins that accumulate in fish and shellfish, mainly bivalves, and force the temporary closure of such production areas, sometimes even for weeks. Spain and Portugal are the European countries where HABs are most reported [7]. To reduce the risk of HABs in seafood and subsequent economical losses, the efficient and timely monitoring of phytoplankton in harvesting zones is needed.

Mycotoxins can be produced by different toxigenic fungi that may occur in a wide range of raw materials and processed foods. In Galicia and Northern Portugal, a similar pattern of toxigenic fungi presence has been observed over the last few decades. These regions are characterized by a high production of maize, wine, and milk, which can be contaminated by mycotoxins from *Fusarium*, *Aspergillus*, and *Penicillium fungi* [8,9]. This is a particularly important concern in the Euroregion as agriculture is of utmost importance for its sustainable development, making it imperative to implement applicable contingency plans in the case of emergencies.

Food allergies are a significant public health concern with increasing prevalence. Allergens can be present in virtually any food, so their control is necessary throughout the agri-food sector, not only during production, but also at the level of transformation and trading. Variations in prevalence among countries are due to differences in consumer genetics in addition to environmental factors, pollen exposure, and eating habits [10]. In Spain, for instance, they affect 3–4% of adults and at least 6% of children. Furthermore, the prevalence of patients attending an allergology consultation for the first time increased from 7.4% in 2005 to 11.4% in 2015, and there is a consensus that the incidence and gravity of cases are increasing [11]. However, in today’s international market, food quality control does not rely on the geographic prevalence of the hazard, but rather on where food may eventually be consumed.

PAHs are hazardous pollutants that easily accumulate in water, soil, and even in living organisms. PAHs can occur as a result of food processing (drying or smoking) and cooking (frying, baking, grilling, etc.) Additionally, PAHs can bioaccumulate in fatty foods through the adipose tissue [12]. It is known that PAHs can produce metabolic alterations, causing cancer tumors and other mutations in animals and humans [13]. In 2005, the European Commission, through the Scientific Committee on Food, established a specific list for food analysis with 15 PAHs [14], 8 of which are included in the US Environmental Protection Agency (US EPA) list.

Listeriosis is a rather infrequent foodborne disease that, nonetheless, has a high case fatality rate (20–30% in immunocompromised people, pregnant women, neonates, and the elderly). Despite significant efforts, the incidence has not decreased in the EU in the past two decades. Accordingly, the incidences in Portugal and Spain (and Galicia) accounted for 21% of recorded cases in the EU [15], although the population of the Euroregion is less than 8% of the total EU population. Seafood, meat, and dairy products were those most frequently found to be contaminated. Epidemiological data from Galicia revealed an average of 1.32 cases per 100,000 persons per year for the period 2009–2015, which was higher than the national average [16].

Commercial analytical methodologies for detecting all the aforementioned hazards have numerous advantages, but also several drawbacks. For instance, in the case of immunological kits used for allergens, mycotoxins, or *L. monocytogenes*, the cross-reactivity with other antigens can lead to false positives or overestimates, whereas the presence of inhibitors in the food matrix or antigen denaturation can lead to false negatives. Therefore, their application is often restricted to some products in which they have been validated [17]. PCR-based methods bypass the need to isolate bacteria, but have shown difficulties in differentiating living cells from dead cells or extracellular DNA, which could lead to false positives or overestimates [18]. Moreover, PCR false positives for allergens can be yielded due to the presence of products of the same species (e.g., cattle or chicken meat would evoke them for cow’s milk or egg, respectively) [19]. Additionally, molecular methodologies demand specialized personnel and expensive, sophisticated equipment.

Chromatographic or mass spectrometry methods can be used to detect PHAs, mycotoxins, allergens, and even bacteria, but they are time consuming and require highly qualified personnel and high-cost equipment. The same limitations also apply to the microscopy methods used to identify toxic phytoplankton, which are especially highly laborious and time consuming. Flow cytometers are also used to identify toxic phytoplankton, but have high cost, are large in size, have insufficient specificity, and mainly cannot be applied for real-time on-site detection. Microbiological methods are time consuming and can provide underestimates as viable, but non-culturable cells are not counted [18]. Last but not the least, all these methodologies are indirect since they rely on sampling, which means representativeness is uncertain in many cases.

Importantly, these methodologies cannot be used for point-of-care detection. In the past two decades, biosensors have emerged as appealing analytical devices for the point-of-care detection of biomolecules of interest in food analysis and food safety. They combine biological components (antibodies, oligonucleotides, aptamers, etc.) with physicochemical detectors (transducer), where the former function as biorecognition elements, while the transducer translates the interactions between biorecognition elements and target analytes into quantifiable signals for measuring analyte concentrations. However, factors such as cost, stability, design, or quality assurance currently limit their commercial availability [20].

The present review addresses the state of the art of analytical methodologies to monitor the aforementioned hazards, which are of high interest for the food industry in the Galicia–Northern Portugal Euroregion. We discuss the pros and cons of commercial and non-commercial methods, with a particular focus on some promising options, particularly biosensors, and future lines of research for point-of-care detection. Lastly, we suggest business opportunities that can arise from the availability of commercial methodologies for each case.

## 2. Toxic Phytoplankton

### 2.1. Characterization of the Hazard

Harmful algal blooms (HABs) are natural phenomena that occur along the coasts of all continents due to the massive proliferation of toxic phytoplankton (cyanobacteria, diatoms, dinoflagellates, and others) in waterbodies. Environmental disturbances, such as climate change, eutrophication, and the introduction of non-native species, are now recognized as factors that may have contributed to the increased frequency and geographic spread of these phytoplankton outbursts [21,22,23]. These events can occur in both large and small areas, depending on the ecology of the species and environmental conditions [24,25]. Of the many thousands of recognized phytoplankton species, a few dozen produce toxins that can accumulate in the tissues of live bivalve mollusks and fish, with direct implications on the mortality of marine mammals, birds, and other animals that depend on the marine food web. These toxins can also have an important effect on humans due to consumption of seafood products [22,26]. On a global scale, marine algae toxins are responsible for more than 60,000 human intoxication incidents per year, with an overall mortality rate of 1.5% [27]. These toxins are molecules of different structures and mechanisms of action, which are classified into different groups according to the syndromes caused, as summarized in Table 1 [23].

The diversity and spatiotemporal distribution of HAB events have been recorded annually in the IOC-ICES-PICES Harmful Algal Event Database (HAEDAT) since 1987 [7,28]. The provided data confirm that the frequency of HABs is increasing globally, and the most common toxin groups in Europe are paralytic toxins (PSP—paralytic shellfish poisoning), lipophilic toxins (DSP—diarrhetic shellfish poisoning), and domoic acid (ASP—amnesic shellfish poisoning) [21]. Portugal and Spain are the European countries in which the most events are reported (669 and 649, respectively) [7]. Thus, monitoring toxic phytoplankton and marine biotoxins in European coastal areas is a priority concern for the EU health authorities. Specific regulations have been established for the different groups of toxins to safeguard public health and minimize the risk of acute poisoning, specifically EC Regulations No. 853/2004 [29], 854/2004 [30], 15/2011 [31], 786/2013 [32], and 627/2019 [33]. These regulations are directly applicable in all European Union Member States.

Marine phytoplankton are the basis of the food web in marine ecosystems upon which all other organisms depend, so the occurrence of HAB events could become a serious problem, as shellfish and fish can accumulate high concentrations of algal toxins [35,36]. This accumulation of toxins is not easily appreciated in affected species and, thus, can be transmitted to humans via different routes, such as consumption of contaminated seafood, skin contact, and swallowing water during recreational activities. Among these routes, the main vectors of human poisoning are seafood, especially filter-feeding bivalve mollusks, such as mussels, oysters, scallops, clams, and cockles, or herbivorous finfish that ingest toxic phytoplankton [22,36].

Seafood is an important source of food and protein for European countries [37]. On average, each European citizen consumes 24.4 kg of seafood products per year, with Portugal (56.8 kg per capita) and Spain (45.6 kg per capita) being the EU countries that have the highest per capita consumption rate [38,39]. Aquaculture is an increasingly important source of seafood protein for the European market [28]. To minimize the risk of seafood contamination and economical losses, programs to monitor the composition of phytoplankton species in harvesting zones are applied. The efficient and timely monitoring of these risk situations, covering a large area, is essential to safeguard public health and the economy, particularly in terms of shellfish and finfish aquaculture [40]. In line with EU policies [41], human health can be safeguarded by monitoring programs that include the detection of toxic phytoplankton as well as the presence of toxins in shellfish. In Portugal, monitoring is implemented by the Portuguese Institute for the Ocean and Atmosphere, whereas in Spain, this is performed by regional government bodies, including the Technological Institute for the Control of Marine Environment in Galicia and the Monitoring Program of Andalusia, which decide and inform the closure and opening of shellfish harvest. Thus, automated phytoplankton monitoring techniques are in high demand as part of a strategy to understand, prevent, anticipate, and mitigate the environmental and economic impacts of HABs.

### 2.2. Methodologies of Monitoring

Certified monitoring procedures for phytoplankton analysis, currently performed by reference laboratories, are based on international standard methods [42] that require the taxonomic identification, counting, and calculation of phytoplankton biovolumes by light microscopy. However, other methods, such as image analysis, pigment analysis by high-performance liquid chromatography (HPLC), flow cytometry, molecular methods, and remote satellite sensing, are also used [43,44,45]. Microscopy and image analysis are technically demanding, time consuming, costly, and predominantly affected by human error as the identification relies on the expertise of taxonomists able to distinguish the morphological differences between different species of phytoplankton [44,46]. Furthermore, the quantification of pico- and nanoplankton by microscopy is often difficult. Spectrophotometric and fluorometric methods, viewed as standards for the quantification of chlorophyll a, are fast and easy, but can only provide a rough estimate of the main groups present in a sample and at a high taxonomic level [45]. The HPLC technique allows the identification and quantification of individual photosynthetic pigments [47], even at low concentrations, but also at a high taxonomic level such that it does not discriminate at the genus or species level. Among these technologies, flow cytometry is the most suitable for high precision quantification and discrimination of phytoplankton cells in a fast and automatic way. It is based on the use of fluorescence and light scattering signals to determine the identity of photosynthetic pigments present in the sample and their size, respectively [44,47]. However, these methods are expensive and require the use of bulky equipment, complex procedures, and highly trained personnel, with some also needing an extraction step and standards for calibration and, in most cases, are difficult to apply in a portable sensing system for in situ detection [43,45]. The need for portable and automated devices for in situ phytoplankton identification has led to the development of some commercial devices, such as FlowCAM, CytoBuoy, Imaging FlowCytobot, or Laser Optical Plankton Counter [44], but they are still bulky and expensive. In addition, there is an effort to analyze phytoplankton based on satellite ocean color imaging with the exploitation of backscattered sunlight, but, at present, the status of the technology is still far from allowing the comprehensive identification and profiling of the diverse phytoplankton community, particularly in coastal areas where problems with HABs are more significant [44,48].

Recent works have developed portable sensors that measure different phytoplankton species based on the fluorescence detection of primary photosynthetic pigment (chlorophyll a) and accessory pigments (carotene, chlorophyll b, phycoerythrin, phycocyanin, and others) present in microalgae and cyanobacteria. Shin et al. developed a handheld phytoplankton sensor that uses different excitation wavelengths (385, 448, and 590 nm), a Si photodiode to detect the emission of fluorescent lights, and a microfluidic chip for sample holding and delivery. This device demonstrated the potential to differentiate a mixture of green algae and cyanobacteria species using a multivariate algorithm with a limit of detection (LOD) for green algae and cyanobacteria of 1 and 4 mg/L, respectively [49]. In addition, Zieger et al. developed a low-cost and miniaturized multichannel fluorometer for the continuous identification and quantification of the major groups of relevant algae (green algae, cyanobacteria, and dinophytes) based on their spectral characteristics, with an LOD of 10 cells/L [43].

Another approach to portable sensors is based on optical image analysis, which allows the individual classification of phytoplankton cells based on their morphology. However, advanced image capture and processing techniques are required. Some works have combined image capture technologies (e.g., FlowCam) with machine learning techniques, particularly artificial neural networks (ANNs), to reduce the complexity of image processing, improve image quality, and increase the performance of phytoplankton species detection [46,50].

Although these new technologies were successful in identifying phytoplankton, they still have some limitations. It is challenging to distinguish multiple species of phytoplankton simultaneously using only fluorescence detection because the fluorescence signals of different species often overlap and interfere with each other. Biofouling and dissolved organic matter in water could become a relevant source of sensor signal variability, requiring regular maintenance. In addition, turbidity correction is needed, especially when the sensors are used in natural conditions [45,47]. Nevertheless, the proof of concept of these technologies is a step forward in the direction of a new generation of phytoplankton-monitoring platforms, with strong potential for the early detection of certain species of phytoplankton that cause HABs with substantial impact in several areas, especially food safety, sustainable management, water quality, and public health. Furthermore, the low cost of these technologies will allow future large-scale deployments, enabling a better understanding of the ocean, with systematic measurements and analyses of toxic (and nontoxic) phytoplankton.

### 2.3. Market, Current State and Prospective

By 2050, the world’s population is expected to reach 9.6 billion (increasing by 34% from today), which means food production and consumption will also increase. To address the increased food demand, alternative food sources must be found. Aquaculture has established itself as a successful technology for providing food resources for human consumption, as evidenced by the important milestone reached in 2014, where the human consumption of fish from aquaculture first exceeded that of wild-caught fish. In fact, the volume of wild-caught fish has remained steady since 1980 while aquaculture production has since experienced a continuous and marked increase [38,39]. However, aquaculture can be particularly affected by HAB events, which cause the contamination of products resulting from this technology [22]. Thus, to ensure the safety and quality of food products from aquaculture, monitoring plans that are especially focused on toxic phytoplankton and contamination by biotoxins have been implemented by regulatory authorities. Although current methods for phytoplankton identification provide high sensitivity (up to a single cell), their limitations have attracted the attention of several companies (such as CytoBuoy) to develop fast and accurate measurement techniques for automated in situ phytoplankton identification. However, they are bulky and expensive. Thus, the range of business opportunities associated with the development of new methods for the identification of phytoplankton with appropriate sensitivity and cost effectiveness is wide, and these methods have the possibility of being offered in many regions around the world.

## 3. *Listeria monocytogenes*-Containing Biofilms

### 3.1. Characterization of the Hazard

*Listeria monocytogenes* is a well-known human bacterial pathogen responsible for listeriosis. Listeriosis is a rather infrequent disease, with an incidence rate of 0.46 per 100,000 people in the EU in 2018 and 2019, but with high case fatality and hospitalization rates, causing more than half of fatal cases by zoonotic agents [15]. The fatality rate is commonly estimated to range between 15% and 30% in pregnant women, neonates, immunocompromised people, and the elderly. However, an even higher severity was reported in a recent cohort study (MONALISA) showing, for instance, that more than 80% of pregnant women experienced major fetal or neonatal complications, and only 39% of patients with neurolisteriosis survived and fully recovered [51].

Approximately 99% of listeriosis cases are due to the consumption of contaminated food [52], generally ready-to-eat (RTE) products containing more than 2000 CFU/g (92% of cases) [53], with the highest incidence of *L. monocytogenes* being found in RTE seafood, followed by RTE meat products [54]. Three microbiological criteria are laid down for *L. monocytogenes* in different RTE foods in the EU (Regulation 2073/2005) [55]. Accordingly, food operators must implement measures to prevent the presence or growth of *L. monocytogenes* in foods. However, the incidence of listeriosis increased from 2008, when it became a notifiable disease, to 2015, remaining stable subsequently. A similar pattern has also been observed in Galicia and Portugal [1,15].

Some factors account for these patterns. Firstly, RTE products are increasingly consumed in industrialized countries due to changes in food consumption—indeed, in social trends—in the past few decades [56]. Data from Statista [57] show that such consumption patterns have also been adopted in Portugal and Spain. Many of these products are minimally processed and must be stored under refrigeration, but *L. monocytogenes* can resist minimal processing and grow at temperatures down to 0 °C, which gives it a significant competitive advantage over other bacteria and can make it particularly hazardous.

In addition, *L. monocytogenes* is found in farms, food processing plants, domestic environments, and in nature, as part of biofilms [58]. Biofilms are much more resistant to harsh conditions than free-living cells and, therefore, much more difficult to remove via sanitation measures, making them more likely to contaminate food. According to EU legislation, operators manufacturing hazardous RTE foods must sample the surfaces of processing areas and equipment for *L. monocytogenes* and correct any deficiencies in sanitation to prevent them from being present in biofilms (Regulation 2073/2005) [55]. However, this is rather challenging as biofilms are rather common in food processing facilities, particularly in hard-to-access points, irregular surfaces, and sites where organic matter tends to accumulate [59], and, although *L. monocytogenes* is not usually a biofilm-forming bacteria, it tends to associate with other microorganisms in polymicrobial biofilms.

### 3.2. Methodologies for Detection

Nowadays, classical culture methods are still used to detect *L. monocytogenes*, specifically ISO 11290-1 [60] and ISO 11290-2 [61], which are the analytical reference methods in EU Regulation 2073/2005 and the Guidelines on Sampling Food Processing Area and Equipment for Detection of *L. monocytogenes* [62].

Surface monitoring using culture methods is reliable, simple, low cost, and highly sensitive, but has several drawbacks [18,63]. Such methods are time consuming, particularly for pathogens such as *L. monocytogenes*, which can only be found at extremely low levels in highly heterogeneous polymicrobial biofilms [58], so enrichment steps are needed. Consequently, it takes several days to provide results, hindering the rapid and immediate application of corrective actions. Furthermore, stressful conditions (nutrient privation, hypoxia, and the presence of antimicrobials) are frequent within biofilms and, consequently, bacterial cells could be sub-lethally injured or enter a viable but non-culturable state, which may prevent them from growing in culture media used for detection. However, they can resume growth and become virulent under some favorable conditions. In addition, bacteria must be detached from surfaces for analysis, which restricts monitoring to a low number of sampling points and makes representativeness uncertain, particularly in large facilities and hard-to-reach sites. Furthermore, many cells are not detached, whereas others are retained within the sampling tools, which, in both cases, prevents them from being detected.

Alternatively, monitoring can be performed by contact plates or even control surfaces containing small-size coupons (e.g., SCH^®^, Eurofins, Barcelona, Spain), which are fixed and left on food industry surfaces for continuous exposure to real environmental conditions. Both systems allow direct sampling but must be removed for ex situ microbiological analysis. Additionally, they can only be applied on flat, smooth, easy-to-access sites and still have most of the drawbacks of classical culture methods.

The development of methodologies that overcome the drawbacks of classical culture methods has long been a major objective for researchers and the industry. As a result, a wide array of commercial kits and scientific instruments can now be used to detect *L. monocytogenes* and other foodborne pathogens, and others are currently in development and seem highly promising. In general terms, three main methodological approaches have been followed.

1. Phenotypic identification systems. Miniaturized biochemical test galleries have been widely used (e.g., API^®^ Listeria, bioMerieux, Marcy-l’Étoile, France). Reading can be automated with instruments, such as VITEK^®^ 2 COMPACT (bioMerieux, Marcy-l’Étoile, France) or BD Phoenix™ AP (Becton-Dickinson, Franklin Lakes, NJ, USA), which rapidly provide results, but the equipment is costly and previous isolation of microorganisms is needed, which entails growing them on culture media and prolongs the time to achieve results (24–48 h). Nowadays, microbial identification is increasingly conducted from peptide mass fingerprinting as determined by MALDI-TOF (time-of-flight) mass spectrometry, such as VITEK^®^MS (bioMerieux, Marcy-l’Étoile, France) or microflex^®^ LRF (Bruker, Billerica, MA, USA) systems [64].

2. Immunological techniques, mostly enzyme immunoassays, based on monoclonal or polyclonal antibodies. Immunoassays, such as VIDAS LMX, LMO2, and LDUO (bioMerieux, Marcy-l’Étoile, France) or Transia Plate (BioControl Systems, Bellevue, WA, USA), have been validated and some can be used on fully automated platforms or microplate processors. Cross-reactivity with similar antigens from other microorganisms, lower sensitivity than culture- or PCR-based methods, and the inability to detect some isolates are major bottlenecks for their widespread adoption [65]. There is also a noticeable commercial offer of simple, fast, and economic flow lateral kits, which provide fast qualitative responses for rapid screening (e.g., Singlepath^®^ L’mono, Merck; Reveal^®^ 2.0 for Listeria, Neogen, Lansing, MI, USA; VIP^®^ Gold Listeria, Merck, Kenilworth, NJ, USA).

3. Molecular techniques, based on the amplification and detection of specific gene markers. Nowadays, there is a wide array of commercial PCR kits, mostly qPCR-based kits, which are highly specific and sensitive, with an LOD down to 1–5 CFU/25 g food [66]. Among these kits, we can find iQ-Check (Bio-Rad, Hercules, CA, USA), SureTect™ (Thermo Fisher Scientific, Waltham, MA, USA), MicroSEQ™ (Applied Biosystems, Waltham, MA, USA), or BAX^®^ (DuPont-Qualicon, Wilmington, DE, USA). Particularly, the BAX^®^ system was adopted for surface monitoring of *L. monocytogenes* in the food industry by the USDA FSIS. There are also commercial kits working under isothermal conditions, with no need for a thermocycler (LoopampTM, Eiken Chemical Co., Tokyo, Japan; 3M Molecular Detection Assay, 3M, Saint Paul, MN, USA).

Unlike these DNA-based approaches, fluorescence in situ hybridization (FISH) techniques allow specific bacteria to be directly located within biofilms through the use of specific oligonucleotide probes. However, they suffer from drawbacks related to issues with poor cell permeability, probe affinity, and target accessibility, particularly in biofilms. The use of peptide nucleic acids and other developments has resulted in increased performance [18]. However, it is hard to foresee the use of microscopic techniques for point-of-care detection of *L. monocytogenes* on food industry surfaces (see below within this section for further clarification).

Both immunological and molecular methods are highly specific, so isolation of the microorganism to be detected in culture media is not needed. However, pre-enrichment, or, alternatively, some pretreatment (e.g., immunomagnetic separation) aimed at separation or concentration, is generally required, particularly in polymicrobial biofilms, where *L. monocytogenes* is found only at low levels. Furthermore, only qPCR following propidium monoazide (PMA) treatment has been successfully applied to distinguish living cells from dead cells or cell debris [17]. However, PMA pretreatment was shown not to work properly in mature biofilms, which would have physically hindered PMA from binding to DNA or the light required for it to intercalate DNA [67]. Moreover, it has been claimed that it does not work well with dead cells with membrane integrity or living cells with injured membranes, which can be particularly relevant for biofilms on surfaces treated with disinfectants [18]. Pre-enrichment has also been used occasionally to bypass this drawback [68]. Recently, direct metatranscriptome RNA-seq and multiplex reverse transcription–PCR amplicon sequencing on MinION (Oxford Nanopore, Oxford, United Kingdom) were developed for real-time multiplex identification of viable pathogens in food, one of them being *L. monocytogenes* [69]. Both approaches seem promising, partly because the MinION sequencer is reported to be rapid, cost effective, portable, and with high-throughput sequencing workflows.

In summary, all these methods are highly specific for *L. monocytogenes*, but require long experimental protocols, specialized personnel, or expensive machinery, which makes them unsuitable for routine use in the food industry and point-of-care detection. To circumvent such drawbacks, the development of portable, high-throughput, and automated biosensing devices has been a major objective over the past two decades, with optical and electrochemical biosensors as the most widely addressed options.

Optical biosensors enable real-time detection via a method that has high specificity, low cost, and is easy to use [70]. Among possible options, plasmonic biosensors have been found to be highly successful in detecting *L. monocytogenes* [71,72]. Different techniques (fiber optics, Raman, fluorescence, and luminescence) have been coupled to plasmonic biosensors to reduce the LOD. These techniques can be applied to solid and liquid samples, reducing time, price, and reagent consumption [73].

Among plasmonic methodologies, surface-enhanced Raman scattering spectroscopy (SERS) is particularly attractive due to its high selectivity and spectral specificity, non-destructive testability, and multiplexing capability [74]. Sensitivity is significantly enhanced by the implementation of noble metallic nanoparticles conjugated with bioreceptors [75], which has allowed SERS biosensors to detect only a few *L. monocytogenes* cells [76,77]. Recently, SERS has even allowed different genoserogroups to be distinguished [78]. However, some pretreatment (filtration, dielectrophoresis, immunoseparation, etc.) is needed for complex matrices to separate or concentrate the organisms to be detected and reduce interference [79].

Electrochemical biosensors have also become interesting alternatives to conventional methods for detecting low levels of bacterial contamination [80]. They are frequently modified with nanoparticles or conductive polymers to increase their analytical characteristics and enhance detection signals [72]. They have been claimed to have advantages over optical sensors, such as being able to work in turbid media and being more amenable to miniaturization (i.e., portability and ease of use), plus requiring simpler and cheaper equipment [71]. Among the possible options, electrochemical impedance spectroscopy has become the most common for detecting foodborne bacteria [72]. Impedance biosensing allows fast response times, making them appropriate for point-of-care detection of biological agents, such as *L. monocytogenes*, in food and the environment [75], and have an LOD as low as 1 log CFU/mL [81].

Nowadays, the main limitations of biosensors are the nonspecific adsorption and decreased sensitivity and selectivity of their application in complex foods that hold substantially high contents of nontarget molecules [75]. The commercialization of biosensors is also currently affected by several issues, such as high cost, short lifetime, and low stability [82].

Biofilm research has also followed a different strategy aimed at monitoring surface sanitation. This main objective has led to the devising of several fast, low-cost, nonspecific stain-based approaches to detect and quantify biofilm biomass. Most of the methods have not been suitable for use in the food industry, but the successful application in food facilities has been possible for some commercial products, such as BioFinder (Itram Higiene, Barcelona, Spain), TBF^®^ 300 (Betelgeux, Valencia, Spain) or Realco (Ottignies-Louvain-la-Neuve, Belgium), which allow rapid biofilm monitoring based on simple visual inspection after spraying them onto test surfaces. However, none of these products report on contamination levels and, more importantly, they do not distinguish viable cells from dead cells or debris biomass. Moreover, BioFinder can only be applied on clean, exposed surfaces and has a relatively high LOD (10^4^ CFU/cm^2^), and TBF^®^s cannot provide adequate detection in the case of porous surfaces.

Interest in the food industry is focused on detecting viable cells to locate bacterial contamination and assess the effectiveness of sanitation. The ATP bioluminescence assay is probably the most widely used method for hygiene monitoring and cleaning validation [83], and there are portable devices commercially available for measuring ATP bioluminescence (e.g., LIGHTNING MVP ICON). Although sample collection is needed, the results are provided in practically real time (seconds or minutes), which allows the quick application of corrective action. Unfortunately, measurements do not differentiate between ATP from bacterial and non-bacterial contamination, and microbial load can be underestimated in mature biofilms or following poorly effective disinfection [84]. Similarly, some methods that measure the formation of colored compounds from bacterial metabolic activity have been tested for the detection and quantification of viable cells on surfaces, such as those using tetrazolium salts or resazurin [85]. Generally, they work well for planktonic bacteria, but the presence of metabolically inactive cells in biofilms makes them difficult to use for monitoring purposes in the food industry and, moreover, the LOD is rather high.

An array of microscopic techniques have also been historically used for biofilm studies, specifically to estimate biomass and visualize the density and distribution of cells and other constituents, in many cases with the help of specific fluorescent markers [86]. In addition, fluorescence microscopy allows viable and non-viable cells to be distinguished and therefore allows the testing of hygiene protocols through the Live/Dead™ Baclight™ Bacterial Viability kit (Thermo Fisher Scientific, Waltham, MA, USA) or the location of specific microorganisms in mixed biofilms using FISH (see above). These techniques are widely used for research and clinical activities, but the need for sophisticated high-cost equipment and highly qualified personnel, along with a small field of view (<0.1 mm), makes it highly unlikely they will become commonly used in the food industry. 

To enlarge the viewing scales and try to obtain a representative view of biofilms, some non-invasive spectroscopic or spectral technologies operating at the mesoscale (a few mm) or macroscale have been applied for the study of biofilms. Among these technologies, optical coherence tomography seems quite promising for the real-time in situ visualization of biofilms [87]. Other spectral alternatives—such as Fourier transform infrared spectroscopy (FTIR); Raman spectroscopy, particularly surface-enhanced Raman spectroscopy; hyperspectral analysis; or nuclear magnetic resonance—also have the potential to monitor surface sanitation, but are currently in an experimental stage, and point-of-care application is still quite challenging and would likely necessitate the development of portable devices [88].

### 3.3. Market Opportunity

It has recently been estimated that the cost of a foodborne outbreak can range from USD 4000 to 2.6 million, which outweighs all costs of prevention and control measures [89]. Outbreaks of listeriosis, in particular, are the costliest. Generally, listeriosis is responsible for over 50% of deaths caused by zoonotic agents in the EU, and hospitalization is required in around 98% of cases [15]. The costs due to listeriosis were estimated at around USD 3200 million in the US in 2018, representing 18% of the total cost burden of foodborne illness [90].

Changes in food consumption habits require that products be less processed and easier to prepare in industrialized countries, in general, and in the Galicia–Northern Portugal Euroregion, in particular. As a result, food production tends to provide an increasing volume of a wide variety of minimally processed ready-to-eat foods, which have been primarily associated with listeriosis. The production of RTE food will continue to increase in the coming years, especially in regions where food production is of socio-economic importance, such as the Galicia–Northern Portugal Euroregion.

In the EU, business operators manufacturing RTE foods must implement control measures to control the presence of *L. monocytogenes* in food and food facilities by legal imperative [55]. However, the incidence of listeriosis has not decreased, but an increase was observed between 2008 and 2015. A similar pattern has been observed in Galicia and Portugal, with an incidence over the average [1,15]. Therefore, *L. monocytogenes* remains a major food safety hazard and its control is a priority in the EU, including in the Galicia–Northern Portugal Euroregion.

None of the current methodologies can be used for point-of-care detection of *L. monocytogenes* in foods nor in food facilities, where it can even persist for long periods [91]. Surface monitoring is vital to adopt corrective measures in sanitation. Consequently, there is a clear business opportunity for the development and commercialization of methods able to rapidly and reliably detect *L. monocytogenes*. In fact, such methods would have a large market niche [77].

## 4. Mycotoxins

### 4.1. Characterization of the Hazard

Mycotoxins are small, toxic chemicals (MW ~700) produced mainly by saprophytic fungi from the *Aspergillus*, *Penicillium*, and *Fusarium* genera under favorable environmental conditions. The most relevant groups of mycotoxins in terms of public health and global economy are aflatoxins (AFs), produced by *Aspergillus*; ochratoxin A (OTA), produced by *Aspergillus* and *Penicillium*; trichothecenes (group A: HT-2 and T-2 toxin; group B: deoxynivalenol) and zearalenone (ZEA), produced by *Fusarium*; fumonisins B1 and B2 (FB1 and FB2), produced by *Fusarium* and *Alternaria*; and ergot alkaloids, produced by *Claviceps* [92]. In general, several mycotoxins can be found in the same food product since a certain fungal species can produce more than one mycotoxin and the same mycotoxin can be produced by different fungal species.

Toxigenic fungi may be present in raw materials or processed food throughout the entire food chain. Foods most contaminated with mycotoxins are cereals and cereal-based foods, animal feed, dried fruits, nuts and seeds, fruit and vegetables, herbs and spices, juices, wines, coffee, and cocoa. These toxins or metabolites can also be found in animal products, such as milk or meat [92,93].

In the Galicia–Northern Portugal Euroregion, the presence of toxigenic fungi has had a similar pattern over recent decades. The two component regions are closely related from a sociocultural and agricultural point of view, with a large production of maize and wine, but also milk and fish; thus, they have common problems. The most prevalent mycotoxins that contaminate maize in Galicia–Northern Portugal come from *Fusarium* infections, namely, trichothecenes, zearalenone, and fumonisins [8,9]. In wines, particularly in red wines, the presence of OTA is frequently detected, while in milk, the presence of AF, specifically AFM1, is of concern due to its toxicity [8,9]. These contaminants are especially relevant in this Euroregion, where agriculture is of utmost importance for its sustainable development, which makes it imperative to implement appropriate emergency plans.

**Figure 2:**
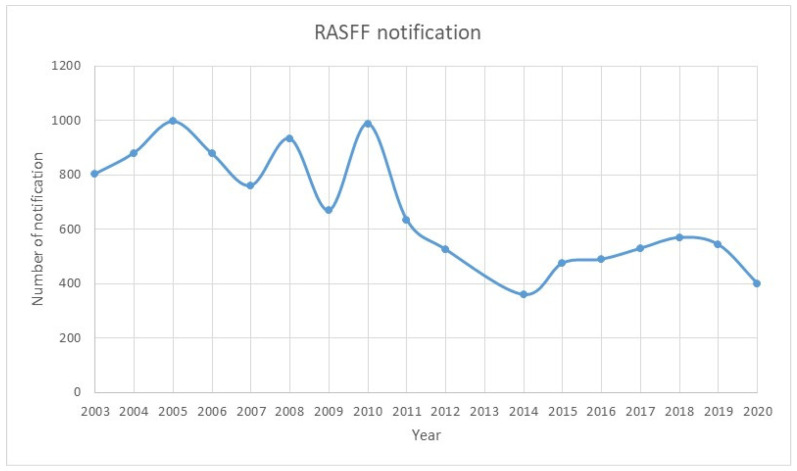
Total notifications for the presence of mycotoxins in food and feed products from European markets according to year.

The pattern of mycotoxin contamination verified in Galicia–Northern Portugal is also reflected in Europe. In the past few decades, the hazard notifications reported in the RASFF (Rapid Alert System for Food and Feed) (Figure 2) related to mycotoxin in food and feed refer mainly to contamination by AFs, followed by OTA, deoxynivalenol, fumonisins, and patulin; the products with the most reports of contamination were nuts, nut products and seeds, followed by fruits and vegetables. The apparent decrease in notification events in the past eight years (Figure 2) can be associated with an increased awareness of the presence of mycotoxins. It is interesting to highlight a decrease of 23% in 2020 compared with the previous year, which could be related to the crisis of the COVID-19 pandemic. However, this cannot be assured with certainty, as a declining trend was observed in recent years. This could be a consequence of both the implementation of more restrictive legislation and the development of increasingly sensitive analytical methods, as we further discuss later in the paper.

Human exposure to mycotoxins may occur through ingestion, inhalation, or dermal contact, though the former is the most common route. The consumption of contaminated agricultural food as well as foods of animal origin that can carry biotransformation products of these contaminants can cause health issues. Mycotoxin can cause a myriad of acute and chronic detrimental health effects, including immunosuppressive, carcinogenic, estrogenic, gastrointestinal, and kidney diseases. The International Agency for Research on Cancer (IARC) classified AFs as carcinogenic to humans (Group 1) and OTA, sterigmatocystin, and fumonisins as possible carcinogens (Group 2B), while trichothecenes, PAT, and ZEA are not classified as carcinogens to humans (Group 3). In addition to causing health issues, mycotoxins have significant economic impacts on numerous crops, particularly cereals, nuts, and coffee, because of yield loss induced by toxigenic fungi and the reduced value of contaminated crops. Measures to control mycotoxins are usually preventive, but not very effective in eliminating the problem, since they are natural contaminants in food.

The growing research on mycotoxins associated with more developed techniques and with lower levels of detection and quantification generated a wave of awareness about the danger they pose, which led to the implementation of exposure limits through food and efforts to reduce their levels to as low as possible [92]. Mycotoxin regulations have been established in more than 100 countries [94], but the maximum acceptable limits differ significantly from country to country. In the EU, regulations for the maximum levels of mycotoxins in food and feed are harmonized among its member countries [94]. Maximum levels and guidance values for mycotoxins in food and feed are set at the European level by Regulation (EC) No. 1881/2006, Commission Recommendation 2006/576/EC [93], Commission Directive 2003/100/EC [95], and Recommendation 2013/165/EU [96].

### 4.2. Methodologies of Monitoring

The analysis of mycotoxins in foods is a challenging subject, since they are present in low concentrations and the techniques employed must therefore be very sensitive and reproducible. To provide faster, cost-effective, and more sustainable analysis, multi-residue techniques are usually chosen. The reference methodologies are based on liquid (LC) and gas chromatography (GC) techniques using diode (UV), fluorescence, or MS detectors [97,98]. They are usually quite expensive, require specialized technology, and do not provide results in situ. Thus, kit-based tests are used as an inexpensive, fast, and easy-to-use alternative to these methodologies.

One of the most widely used methods for the detection of mycotoxins is the ELISA (enzyme-linked immunosorbent assay), which is based on the use of antibodies and has been approved as the official method by the AOAC (Association of Official Analytical Chemists). Other commercially available tests include lateral flow immunoassays (LFIA), fluorometric immunoassays (FA), and quantum dots (QDs).

Notwithstanding the accuracy of the analytical method, sampling represents a crucial step in the assessment of these contaminants. Thus, Regulation (EC) No. 401/2006 [99] and Regulation No. 691/2013 [100] set the methods of sampling and analysis for the official control of mycotoxins in foodstuffs and feed. Most of the analytical procedures include a pretreatment step, where mycotoxins are solvent extracted to obtain an extract that is further purified to remove unwanted co-extracted matrix components and, finally, separation and detection take place.

Of the chromatographic methods, the Quick, Easy, Cheap, Effective, Rugged, and Safe (QuEChERS) procedure is commonly used for extraction [98]. After extraction and purification, chromatographic separation is commonly performed using LC. Then, detection is frequently conducted by ultraviolet (UV), fluorescence (FLD), or MS/MS. The latter provides highly selective and sensitive determinations, resulting in precise and multicomponent outcomes in a short period of time. Electrospray ionization MS/MS is usually operated in a positive mode in dynamic multiple reaction monitoring, using two transitions (quantification and confirmation) [98,101]. Separation by GC methods require prior derivatization to counteract the low volatility and high polarity of many mycotoxins [102,103]. In the manner of LC–MS/MS, GC–MS methods allow the reliable and sensitive determination of multi-mycotoxins in one single run.

ELISA methods represent a commonly used immunoassay method to rapidly monitor mycotoxins and are routinely used by agri-food laboratories. Most ELISA kits for mycotoxin analysis are based on a competitive format, which is a strategy usually applied when the antigen is small and only has one antibody binding site (epitope) [104]. A key step is the immobilization of the sample antigen (mycotoxin), either directly to microwells or indirectly via a detection antibody specific for the target mycotoxin.

Commercial kits are generally endowed with suitable selectivity and sensitivity, require little sample preparation, and allow high operative yield. However, they are time-consuming, with incubation times of 1–2 h, although some commercial kits—for AFS, DON, FUM, OTA, T2, and ZEN—work with incubation periods of as little as 15 min. In addition, there is often an overestimation of mycotoxin levels due to cross-reactivity with the analogs in question. False positive results are also common due to the matrix effect [105]. To eliminate this drawback, most kits have specifications for a single type of matrix, which makes them disadvantageous for a wide application. Another limitation of these kits is that they require specific reading systems that are exclusively marketed by the brands that produce them.

LFIA or membrane-based test strips are commercially available in the form of kits and provide qualitative or semi-quantitative results. They are simple to perform and, as a rule, fast and capable of generating results in minutes [106]. However, problems arise as a result of the rapid saturation of the nitrocellulose membrane used, which can lead to significantly inaccurate results [105].

FA tests also typically require sample extraction followed by extract purification through solid-phase extraction or immunoaffinity columns before binding to a fluorescent molecule and subsequent specific reading on a fluorometer. FA provides indirect measures based on the competition between the analyte and the tracer (fluorescent derivative of analyte). The level of the analyte is determined by measuring the reduction in the fluorescence polarization value, which is determined by the reduction in the level of tracer molecules able to bind the antibody [107]. Therefore, drawbacks include long analysis time and high costs [105].

QDs are photoluminescent nanomaterials with remarkable optical properties, including enhanced photostability, high quantum yields, absorption over a wide range of wavelengths, and narrow and symmetric emission bands that can be adjusted during the synthesis process according to the size of the quantum dot and expected use [108]. These nanomaterials have been used in the development of sensor devices for the determination of multiple analytes, both organic and inorganic, based on the modulation of their fluorescence (inhibition or enhancement of fluorescence intensity, bathochromic or hypsochromic shift) and have high application potential for the determination of mycotoxins in food samples. The main problem associated with the use of QDs is their low selectivity, especially in samples with complex matrices. Therefore, several strategies have been developed to overcome this problem, namely, bioconjugation with antibodies and aptamers, functionalization of the surface of the QDs with molecularly imprinted polymers (MIPs), and combination of different QDs with other nanoparticles for the implementation of donor–acceptor pairs in resonant fluorescence energy transfer (FRET) processes [106]. The conjugation of QDs with different types of molecules makes it possible to take advantage of their optical properties while maintaining the high specificity and molecular recognition capacity provided by functionalization molecules.

### 4.3. Market, Current State and Prospective

Currently, rapid tests for mycotoxin analysis are competitive, with several companies marketing similar products, most of them based on the ELISA methodology. However, new methods that overcome ELISA drawbacks are needed, specifically in the replacement of antibodies by synthetic binders to obtain cheaper kits that allow the detection of mycotoxins by portable devices. Additionally, there is a growing tendency toward the miniaturization of portable devices, which represents a promising market opportunity. However, currently available portable devices do not yet seem to offer sufficient guarantees to potential users, and the positive results obtained must always be confirmed by more precise methods, such as LC–MS/MS.

New biosensors that detect multiple mycotoxins in any food matrix, including processed foods, are challenging for scientists and desirable for consumers. The market expects these new biosensors to provide reliability, sensitivity, selectivity, specificity, and robustness comparable to the conventional analytical system (LC–MS/MS).

## 5. Food Allergens

### 5.1. Characterization of the Hazard

The term food allergy is used to describe an adverse immune response to foods [109]. Food allergies are a significant public health concern, and their prevalence is increasing worldwide, affecting between 3% and 4% of adults and at least 6% of children [109,110,111]. The common mechanism among food allergies is the breakdown of clinical and immunological tolerance against ingested foods, which may or may not be mediated by immunoglobulin E (IgE). Typical symptoms include alterations in the skin, respiratory tract, and gastrointestinal tract as well as cardiovascular alterations. In severe cases, the response is in the form of anaphylaxis, which can be fatal due to hypovolemic shock and respiratory compromise [110]. In addition to allergies, different foods can induce food intolerance, which has a non-immunological base, such as non-celiac gluten sensitivity and lactose intolerance, which are likely the most important examples [112]. Some foods can trigger different pathologies through different pathogenic routes; for instance, wheat can induce celiac disease, allergy, and intolerance [113]. The main way to prevent food allergies is to avoid foods containing the allergen, but this is not easy since some allergens may be present as unsuspected food ingredients, as technological aids in food processing, or after cross-contamination during the production, storage, elaboration, or distribution of foods.

Although there is an increasing number of foods that can cause food allergies, there is a group whose presence must be relayed to consumers. This group is stated in European legislation (Regulation (EU) No. 1169/2011 of the European Parliament and of the Council, which applies to both sides of the border), and includes cereals containing gluten, crustaceans, eggs, fish, peanuts, soybeans, milk, nuts, celery, mustard, sesame seeds, sulfur dioxide and sulfites, lupin, and mollusks [114]. This list includes IgE- and non-IgE-mediated food allergens also in addition to those causing celiac disease. About 75% of allergic reactions among children are due to eggs, peanuts, cow milk, fish, and various nuts. About 50% of allergic reactions among adults are due to fruits of the latex group and of the Rosaceae family, vegetables of the Apiaceae family, and various nuts and peanuts [10]. Food recalls related to allergens are mainly due to undeclared presence or incorrect labeling and can be consulted on the RASFF portal [115]. According to the 2019 RASFF Annual Report, there were 194 notifications related to allergens. Milk, gluten, and soy were the most commonly reported allergens, and cereals and bakery products were the most often notified food types [116].

The main source of allergens is the food itself, although it is the less common that the situation is related to an outbreak or clinical cases, which are associated with blends where the allergen is a minor component or the use of allergen-containing foods or food derivatives as technology adjuvants in the food industry (for instance, thickening agents derived from flour or legumes). Allergy to food additives and preservatives is generally uncommon [107]. Another major concern is cross-contamination, which can occur at all steps of the farm-to-fork chain. This may happen mainly by surface contact between allergenic and non-allergenic food and by allergen transport through air. The frequency of cross-contamination as a cause of accidental exposures to allergenic foods is unknown, and it was proposed that published reports only represent a small fraction of the actual situation [117]. Finally, novel foods may represent novel hazards, or even new sources of already known hazards; for instance, the potential cross-reactivity between insects, which have been considered as the new livestock, and crustaceans and mites has been proposed, and insect consumption therefore poses a risk for people allergic to crustaceans and mites [118,119].

### 5.2. Methodologies of Monitoring

Allergen detection in foods is a challenging task due, in part, to the complexity and variety of the food matrix, which can affect recovery, and the fact that there is no reference concentration for most allergens [114]. On the other hand, there is a need for harmonization in analytical methodology and validation, expression of results, reference materials, and proficiency testing. Regarding the analytical methodology, there are direct methods for detecting the target allergenic protein and indirect methods that detect the genes encoding the allergenic protein by PCR. Among the direct methods, LFIA is rapid, easy to use, and does not require trained personnel, which make it advantageous for use in factories or restaurants, giving a qualitative result. ELISA is commonly used for routine accurate and sensitive quantitative analysis on a broad range of food matrices and environmental samples. Complex samples, as well as highly processed foods, may have concerns related to some extent of protein denaturation, which can affect reactivity and other quality parameters of method performance that must be evaluated [120]. MS combines the strength of the molecule separation of LC and the specificity and sensitivity of mass spectrometry. One of the main advantages is that MS can detect allergens derived from highly processed foods, such as from fermentation, where the allergenic protein has been altered, which can lead to negative results when using other methods. Since the identification does not rely on antibodies, it can detect denatured proteins, although modified proteins do not often show allergic effects. Indeed, multiplexing is easier than in immunological methods [121]. The main problem is that it requires expensive equipment and trained personnel. Allergens are analyzed based on specific peptides obtained after enzymatic degradation that are then isolated by LC and subsequently identified by MS. MS has been combined with proteomics, allowing it to overcome several ELISA limitations related to isoform diversity, post-translational modifications, and other structural changes during food processing and degradation in the gastrointestinal tract [122,123].

Particularly, different proteomics approaches have been tested: selected/multiple reaction monitoring (SRM/MRM), data-independent acquisition (DIA) method, and the sequential window acquisition of all theoretical fragment ion spectra MS (SWATH-MS). Additionally, 1-DE and 2-DE followed by Western immunoblotting, MALDI-TOF/TOF, and, more recently, LC–MS/MS, have also been used. In general, the use of proteomics and transcriptomics has been more focused on vaccine design and the identification of cross-reactivity and new allergens, as well as on the development of strategies to reduce allergenicity, rather than for analytical purposes [123].

Among the indirect methods, PCR assays detect genes encoding the allergenic protein, but not the protein itself. There are several international guidelines for sample preparation and DNA extraction [123,124,125,126]. Since DNA is much more stable than proteins, protein changes due to food processing or during extraction methods, leading to false negative results in immunological detection methods, are not of concern in nucleic-acid-based detection. The use of PCR methods is dependent on several aspects: establishing the LOD, DNA quantity, the design of reference material, how to transform the DNA result into mg of protein, and the ring test of laboratory performance. Other indirect tests are assays that assess cleaning and assume that the prevention of cross-contamination is only reliant on proper cleaning. Among this group, some assays detect the presence of proteins (without discriminating among allergens and non-allergens: Biuret, Bradford, and BCA colorimetric tests), whereas others, such as ATP detection tests, do not detect protein, but rather the cleanliness level.

Each method has advantages and disadvantages regarding speed, accuracy, and ease of use. Direct methods are preferable for allergen warning, but depending on the situation, a combination of different methodologies may be advisable. The main advantages of ELISAs are that they are quantitative, fast, and easier to use than PCR. They may present issues related to the matrix effect, sensitivity, and specificity. The extraction step may be a major concern, mainly in highly processed foods, since changes in the target antigen may affect antibody recognition, leading to false negatives, whereas cross-reactivity may lead to false positives. In addition, some components of the food matrix may alter the antigen–antibody reaction and hence induce false negatives.

RT-PCR is sensitive and specific, but it presents higher needs in terms of specialized personnel and equipment and produces qualitative results. Compared to proteins, DNA is more stable against heat or pressure, but some highly processed food (vegetal oils, gelatin, and starch) may lack sufficient DNA quantities for analysis. Several commercial kits for foodborne allergen analysis based on RT-PCR already exist, including for crustaceans, fish, mollusks, celery, lupine, mustard, oak celery, gluten, hazelnut, peanut, soy, milk, and buckwheat. Indeed, there are several multiplex kits for the simultaneous detection and differentiation of wheat, barley, and rye and for different tree nuts (macadamia, Brazil, and pecan nuts), with an LOD (ppm range) similar to that of commercial ELISAs [119].

Other analytical technologies are under development, but with scarce commercial presence. These include surface plasmon resonance (SPR) [127,128,129] electrochemical affinity biosensors [130]; the use of MIPs [131], biosensors, and nanoparticles, such as gold, carbon, and graphene [132]; and fluorescence-based methodologies [133]. The use of biosensors is an appealing alternative to instrumental analysis in food control, although there are currently few, if any, commercial applications for allergen control. A different approach for allergen control is devices intended for personal use by individual patients. Examples of this are the Nima Sensor, Allergy Amulet, and integrated exogenous antigen testing (iEAT) pocket detectors for different allergens, such as gluten, peanut, hazelnut, milk, or eggs [134,135].

### 5.3. Market, Current State and Prospective

The market demand for food allergen testing is small compared to other types of food analysis, but it is in continuous growth, driven by the increase in both consumer concern and the prevalence of food allergies. European legislation specifically mentioned 13 food allergens, but there are about 170 foods that may induce food allergy. Europe is expected to be one of the world’s regions with the fastest-growing market demand for allergen methods, due to the high standards of food quality and safety in the region. Currently, ELISA is the main established methodology, but other techniques that overcome its weaknesses, such as MS, have a promising future. Commercial ELISA kits are available for almost all commonly labeled food allergens, and they work at low LOD levels, among 0.5–20 ppm or even lower, with similar analytical performance in terms of sensitivity, selectivity, reproducibility, total assay time, etc. [121]. In a comparable way, several commercial lateral flow kits are available for all common foodborne allergens, and some have even been validated. These commercial kits are qualitative, with an analysis time ranging from 5 to 120 min depending on the target, brand, and extraction protocol, and their LOD is at the ppm level [121].

On the other hand, there is also growing interest in devices intended for consumers’ personal use in terms of market opportunities. In this sense, no significant differences among the Euroregion and the whole Europe should be expected.

## 6. Polycyclic Aromatic Hydrocarbons (PAHs)

### 6.1. Characterization of the Hazard

Polycyclic aromatic hydrocarbons (PAHs) are hazardous pollutants that easily accumulate in water, soil, and even living organisms. Food contaminants can typically be produced as a result of food processing (drying or smoking) and cooking (frying, baking, grilling, etc.) or bioaccumulated in fatty food via the adipose tissue [12]. It is known that PAHs can produce metabolic alterations, giving rise to cancer tumors and other mutations in animals and humans. Since 2005, the European Commission, through the Scientific Committee on Food (SCF), established a specific list of compounds to be included in food analysis that includes 15 PAHs, 8 of which are included in the US EPA list [14].

PAHs are organic molecules characterized by two or more fused aromatic rings comprising over 200 compounds [136]. PAHs can be classified into either light or heavy compounds depending on the number of aromatic rings, namely 2–3 or 4–6 rings, respectively. PAHs can originate from either natural or anthropogenic sources. Bushfires, hydrothermal processes, and volcanoes are the main sources of natural emission that have been identified. In terms of anthropogenic sources, the incomplete combustion of organic matter, such as coal, wood, or fossil fuels, has been identified as the main cause [137].

Different national and international organizations have evaluated the occurrence and toxicity of PAHs, such as the European Food Safety Authority (EFSA), the US EPA, the Joint FAO/WHO Expert Committee on Food Additives (JECFA), and the IARC. Since 2003, the EU has followed the recommendations and advice of EFSA; prior to this, the EU was advised by the SCF.

In 1970, the US EPA proposed a priority list of 16 PAHs based on their occurrence, toxicity, and potential human exposure. In 2002, the EU, through its former advisor SCF, identified 15 PAHs (see Table 2), with just 8 of them included in the US EPA list, as molecules with the potential to produce genotoxicity and/or carcinogenic effects in animals and humans. In 2005, JECFA proposed including benzo[c]fluorine in the list of PAHs.

The IACR assessed the toxicity of the PAHs, classifying them into three different groups: carcinogenic to humans (group 1), probable or possible human carcinogens (groups 2A and 2B, respectively), and not classifiable (group 3) [136]. On the other hand, the SCF proposed up to three different indicators to monitor the occurrence of carcinogenic PAHs: (i) determination of benzo[a]pyrene due to its carcinogenic, mutagenic, and teratogenic properties, although this indicator was later amended since re-evaluation studies from JECFA revealed the presence of other carcinogenic PAHs in foods that were negative for benzo[a]pyrene; (ii) determination of the content of a selection of four PAHs (PAH4, namely, benz[a]anthracene, benzo[b]fluoranthene, benzo[a]pyrene, and chrysene); and (iii) the occurrence of eight different PAHs (PAH8, being benz[a]anthracene, benzo[b]fluoranthene, benzo[k]fluoranthene, benzo[ghi]perylene, benzo[a]pyrene, chrysene, dibenzo[a,h]anthracene, and indeno[1,2,3-cd] pyrene). Different studies revealed that the monitoring of the four molecules (PAH4) gives similar results to PAH8 since, most of the time, when analyzing PAH 4, the other four compounds from PAH8 are detected. The current Commission Regulation (EC) No. 1881/2006, setting maximum levels for certain contaminants in foodstuffs, partially amended by Commission Regulation (EU) No. 1327/2014 regarding the maximum levels of polycyclic aromatic hydrocarbons (PAHs) in traditionally smoked meat and meat products and traditionally smoked fish and fishery products, is directly applicable in both Spain and Portugal.

PAHs may be formed and released as a result of incomplete combustion or pyrolysis of organic matter, such as during forest fires as well as during industrial processes or other human activities, including exhaust from motor vehicles, petroleum refineries, heating in power plants, and tobacco smoke as well as during food processing, preparation, and cooking. In the pyrolysis reaction, radicals react with alkanes, alkenes, and aromatic acids to create PAH ring structures. Owing to health risks, the European Commission has launched efforts to decrease the PAH concentrations in food, especially through strategies to control the processes that induce their formation in addition to monitoring their concentration.

PAHs are ubiquitous in the environment, and their presence in food can be attributed to diverse pathways that include both natural (as environmental) and synthetic sources (e.g., cooking practices and industrial food processing) [136,138]. Typically, these contaminants can be present in raw materials due to the environmental contamination of the air by deposition on crops, contaminated soils, and waters. Furthermore, when contaminated vegetation is used to feed livestock, PAHs can accumulate in products of animal origin, such as milk and its derivatives [139]. Remarkably, the number of wildfires has markedly increased in Mediterranean Europe, including in Spain and Portugal. Notably, the potential environmental contamination originating from anthropogenic fires may be particularly important in Galicia, as it is by far the Spanish region most affected by wildfires, both in absolute number of fires and in terms of affected area [140]. Wildfires can cause several impacts on downstream aquatic ecosystems as a consequence of environmental contamination by PAHs [141,142]. Therefore, the presence of these pollutants in the estuarine and oceanic systems is a global problem and a serious concern for human and environmental health due to the transfer of water to fresh and marine invertebrates. For instance, as filter feeders, bivalve shellfish can accumulate PAHs from contaminated seawater and sediment. The bioaccumulation of PAHs and their associated toxic effects in such marine organisms and onward transmission to humans via the food chain is a major concern. In this context, Galicia is the second-largest mussel producer in the world, with a production that has surpassed 250,000 tons annually [143]. Besides mussels, clams, oysters, and cockles are also important economic drivers in the region, having consolidated a highly profitable stable market for Galicia [144]. This has prompted the administrative authorities, through the Technological Institute for the Monitoring of the Marine Environment in Galicia (INTECMAR), to control and ensure the quality of the shellfish produced on its coast. In this framework, Rodil and collaborators analyzed the presence of a variety of persistent organic pollutants, including PAHs, and determined that this family was found at higher concentration levels. The concentration of PAHs in this study ranged between 6.8 and 318 ng/g dry weight, in agreement with the results reported from other marine environments [145].

Industrial and domestic food preparation, such as smoking, drying, roasting, barbecuing, or frying, are recognized as important sources of PAH contamination. Other factors, such as packaging materials, can also be sources of PAHs [146]. The presence of PAHs in vegetable oils can also originate from the smoking and drying processes used to dry oil seeds. Smoked products and certain cooked meat products, such as flame-grilled burgers, as well as dried foods, including spices and plant- or algae-based supplements, can be susceptible to PAH contamination [146]. Generally, owing to their usually higher concentration of PAHs, smoked fish and meats and barbecued foods may represent a significant health risk when these foods comprise a large part of the diet. Different levels of PAHs are generated in food products depending on factors, such as the time and temperature of the heat treatment, distance to the heat source, type of fuel used, fat content of the food, and utilization of smoke in food processing [147]. The contamination with PAHs through intense thermal processing may be due to their direct deposition from smoke. Several studies have shown the presence of PAHs in traditionally smoked foods from Galicia and Portugal [148,149,150,151,152], highlighting that their chemical safety must be assured through the optimization of the smoking regime. This is highly remarkable, as smoked meat products represent a significant part of the human diet in Galicia and Portugal owing to their high nutritional value and high level of production.

### 6.2. Methodologies of Monitoring

The monitoring of PAHs in food or environmental samples may require the use of different techniques due to the complexity of these matrices. The conventional analytical techniques employed in PAH determination are GC/MS—including chemical ionization MS, ion trap MS, MALDI-TOF, and isotope-ratio MS (IRMS)—and HPLC coupled with fluorescence or ultraviolet detection (HPLC/UV) [136].

Conventional techniques present different advantages, such as in terms of their accuracy, reliability, or sensitivity, but they also have certain disadvantages or limitations that make the development of novel or more user-friendly techniques highly desirable. For instance, as generally accepted drawbacks, we highlight the following: (i) they typically require a separation or extraction process that involves using large volumes of organic solvents, (ii) the inefficiency of the previous process could lead to loss of analytes, and (iii) they are time consuming, and a trained technician is required.

Particularly, HPLC presents selectivity issues that prevent achieving a low LOD for all PAHs due to the limited peak capacity of the columns. Alternatively, GC methods present stronger sensitivity and selectivity combination, especially when applied in tandem with MS. Therefore, although the HPLC and GC methodologies satisfy the legislation requirements, alternative methods have been proposed that are less time consuming, inexpensive, and have improved sensitivity. Among the different analytical techniques, we can highlight fluorescence spectrophotometry, SERS, and electrochemistry [153].

Electrochemical-based PAH sensors have been intensively developed in consideration of the relatively low cost, simplicity, and portability of the instrumentation, as recently reviewed by Comnea-Stancu et al. [153]. Among the different measurable parameters, potentiometry, voltammetry, coulometry, electrical conductance/electrochemical impedance spectroscopy, and voltammetric methods have become the most popular [154].

Modification of the electrodes, typically made of gold, glassy carbon, or indium tin oxide (ITO), with either organic molecules, polyelectrolytes, mesoporous silica, conductive polymers (i.e., polypyrrole or polyaniline), or graphene oxide, allows the fabrication of selective electrochemical sensors for a specific PAH analyte. Additionally, electrochemical sensors that have been combined with other techniques, such as immunoassays [155] or MIP [156], show a high selectivity and sensitivity, thereby allowing the analysis of multiple PAHs on a single platform.

Since its discovery in the 1970s, SERS spectroscopy has become a powerful analytical technique for the ultrasensitive detection of molecules [157]. The unique Raman scattering spectral features with narrow peaks make SERS ideal for multiplexing. SERS spectroscopy is based on the enhancement of the inelastic Raman scattering signals characteristic of an analyte when it is close to a plasmonic metal surface. Unfortunately, the lack of metal affinity of the analyte of interest could limit their effective detection. To circumvent this limitation, the plasmonic substrate can be surface modified with different porous materials as potential and selective adsorbents for the analyte. Additionally, the porous coating would generate sieving effects, reducing potential interferences in complex matrices. Bodelon et al. [158] proposed the modification of a gold nanostructure substrate with pillar [5] arenes for the quantitative, label-free, and multiplex SERS detection of three PAHs based on host–guest hydrophobic and π–π interactions. Similarly, Du et al. [159] proposed a coating with polydopamine that, through π–π interactions, acts as a scaffold for PAH detection. As previously reported for electrochemical sensors, Castro-Grijalba et al. [160] proposed a SERS-based molecularly imprinted plasmonic sensor for highly sensitive PAH detection through the synergistic combination of MIP thin film with a nanostructured plasmonic substrate. The right choice of the PAH template molecule gives rise to a sensor with high selectivity and enables its detection in the nM regime.

### 6.3. Current and Prospective Market State

The detection of PAHs in environmental concentrations as well as the understanding of their biological effects and health risks are areas of research that are currently hotspots. Future research should be devoted to (i) establishing precise and high-efficiency analytical methods for PAH detection in environmental concentrations; (ii) the development of unambiguous animal model studies to understand biological effects in vivo; (iii) the development of unambiguous in vitro models; and (iv) introducing omics methodologies in risk assessment, which can be used to identify environmental exposures related to health and disease as well as epidemiological investigations and biomarker detection [161]. Current research is underway to improve the LODs for field application, as well as to improve current analytical methods. For SERS to be applied in the detection of PAHs, the low affinity of their apolar aromatic ring structure for metallic surfaces must first be overcome. To tackle this challenge, different approaches have been developed to functionalize the surface of plasmonic nanostructures to promote the binding of target analytes. Several studies have demonstrated the feasibility of SERS for detecting and quantifying PAHs with high sensitivity and in a multiplexing format, demonstrating the high potential of this analytical technique for the detection of PAHs in environmental samples [160,162].

## 7. Conclusions

Food production is an important socioeconomic pillar in Galicia–Northern Portugal, where HABs, PAHs, mycotoxins, allergens, and *L. monocytogenes* have been identified as relevant hazards for foods produced in the Euroregion. Despite the establishment of legal regulations to ensure food safety and the current substantial analytical capacity to detect food safety hazards, these hazards occasionally continue to be present at unacceptable levels in food products. Therefore, it seems evident that controlling and monitoring such hazards is challenging, especially for the small- or medium-sized companies that abound in the Euroregion.

Modern consumers are demanding more natural, healthier, and additive-free foods that are also easier to prepare or even ready-made. As a result, the production of a wide variety of ready-to-eat food—in many cases, minimally processed—has been increasing in recent decades, and this trend is expected to continue in the future [57]. Minimal processing and the lack of a microbicidal step before consumption increase the risk of *L. monocytogenes*, particularly in fish and meat products [14,52]. Processed foods often have multiple ingredients and might contain various allergens. As a result, an increasing number of foods can cause allergies. Moreover, fraudulent activities can increase the complexity of allergen detection.

In addition, anthropic activities will continue to release pollutants into the environment in the future (e.g., PAHs), particularly in highly populated areas, such as much of the coastal areas of the Euroregion. Moreover, global warming and climate change can enhance the emergence of food safety issues, such as an increased frequency of HABs and changes in qualitative and quantitative patterns of mycotoxin production.

Such hazards may come from natural sources, but also from the food production environment, anthropogenic activities, or even food processing. Thus, food business operators and food safety authorities must control and monitor such hazards throughout the entire food chain, both now and in the future. Although the currently available methods of analysis provide high sensitivity, specificity, and accuracy, there is a need for improvement in terms of affordability, size, ease of use, and portability. Indeed, point-of-care analytical methodologies that can be used at any production stage are increasingly needed, but none are available yet. Such methodologies would allow an enhanced management and sustainable exploitation of resources, benefitting decision makers, researchers, companies, and society in general. Therefore, there are clear business opportunities for the development and commercialization of methodologies that detect such hazards rapidly and reliably, particularly analytical handheld devices that boast continuous use, low cost, and rapid response and do not require sample preparation while guaranteeing adequate levels of detection.

Different biosensors devices for point-of-care detection of hazards with high sensitivity, specificity, and accuracy are currently being developed. Most importantly, nanotechnology has opened new avenues for the development of robust and reliable functional nanomaterials, which have been applied for sensing foodborne hazards. Therefore, a new generation of miniaturized monitoring strategies for automated in situ detection is expected. This appears to be a priority considering health and economic interests, with a huge impact in several areas, especially in food safety, water quality, pollution control, and public health. Among the developed devices, SERS-based nanostructured sensors are especially promising for the detection of several hazards, such as PAHs, allergens, or bacterial pathogens (e.g., *L. monocytogenes*), as they have demonstrated multiplexing capabilities and high sensitivity below regulative limits. Moreover, the existence of portable Raman instruments could pave the way for on-site detection. However, the need for standardization and validation remains a significant challenge, particularly if such instruments are to be adopted by regulatory agencies.

Despite the importance of monitoring programs for the management of marine activities and the mitigation of HAB impacts, methods for toxic phytoplankton identification are too slow for high temporal and spatial monitoring. Thus, low-cost automated phytoplankton monitoring techniques are in high demand. Microflow cytometry has shown excellent ability for the rapid automated quantification and discrimination of individual phytoplankton cells.

The development of innovative technology to generate new and integrated knowledge about the prevalence of food hazards will have a clear impact on the agri-food industry, allowing producers to ensure food security and safeguard public health.

## Figures and Tables

**Table 1:** Human syndromes and symptoms from HAB toxins and causative phytoplankton species. Adapted from [23,34].

Type of Poisonings	Toxins Produced	Causative Organisms	Symptoms
**PSP** **(Paralytic Shellfish Poisoning)**	Saxitoxins	*Gymnodinium catenatum,* *Pyrodinium bahamense*	Potentially fatal (8.5–14%), death occurs within 24 h. In nonlethal cases: tingling, numbness, ataxia, giddiness, drowsiness, fever, rash, and staggering.
		*Alexandrium* spp. (e.g., *A. minutum,* *A. tamarense,* *A. ostenfeldii*)	
		Marine cyanobacteria (e.g., *Anabaena, Aphanizomenon, Planktothrix, Lyngbya, Cylindrospermopsis*)	
**ASP** **(Amnesic Shellfish Poisoning)**	Domoic Acid	*Pseudo-nitzschia* spp. Seriata group (cell width >3 µm)	Within 24 h: nausea, vomiting, abdominal cramp and diarrhea. Within 48 h: neurological symptoms, such as dizziness, headache, seizures, disorientation, short-term memory loss, respiratory difficulty, and coma.
		*Pseudo-nitzschia* spp. Delicatissima group (cell width <3 µm)	
**DSP (Diarrhetic Shellfish Poisoning)**	Okadaic Acid	*Dinophysis* spp. (e.g., *D. acuta*, *D. acuminata*, *D. fortii*, *D. ovum*) *Prorocentrum* spp. (e.g., *P. lima*, *P. cordatum)*	Diarrhea, nausea, vomiting, abdominal cramps, and chills.
**AZP** **(Azaspiracid Shellfish Poisoning)**	Azaspiracid	*Azadinium spinosum*	Diarrhea, vomiting, and abdominal cramps.
**CFP (Ciguatera Fish Poisoning)**	Ciguatoxin/Maitotoxin	*Gambierdiscus toxicus*, *Prorocentrum* spp., *Ostreopsis* spp., *Coolia monotis*, *Thecadinium* sp., *Amphidinium carterae*	Initially diarrhea, vomiting and abdominal pain, followed by neurological dysfunction or temperature sensation, muscle aches, dizziness, anxiety, sweating, numbness, and tingling of the mouth and digits.

**Table 2:** Structures, names, and toxicity group of PAHs according to the EU IACR toxicity group.

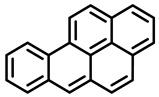	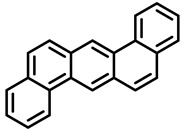	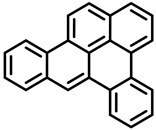
Benzo[a]pyrene	Dibenz[a,h]anthracene	Dibenzo[a,e]pyrene
Group 1	Group 2A	Group 3
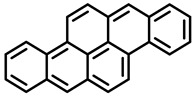	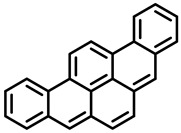	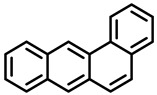
Dibenzo[a,h]pyrene	Dibenzo[a,i]pyrene	Benzo[a]anthracene
Group 2B	Group 2B	Group 2B
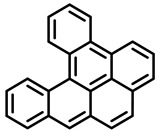	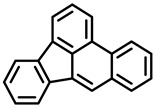	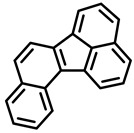
Dibenzo[a,l]pyrene	Benzo[b]fluoranthene	Benzo[j]fluoranthene
Group 2A	Group 2B	Group 2B
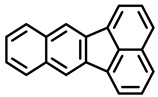	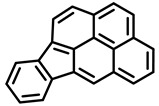	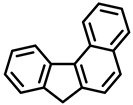
Benzo[k]fluoranthene	Indeno[1,2,3-cd]pyrene	Benzo[c]fluorene
Group 2B	Group 2B	Group 3
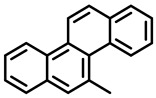	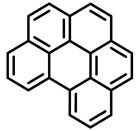	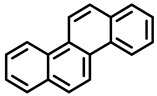
5-Methylchrysene	Benzo[ghi]perylene	Chrysene
Group 2B	Group 1	Group 2B
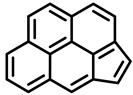		
Cyclopenta[cd]pyrene		
Group 3		
